# The Use of the Screw in Regulating Teeth

**Published:** 1883-10

**Authors:** William H. Trueman

**Affiliations:** Philadelphia, Pa.


### THE USE OF THE SCREW IN REGULATING TEETH.

BY WILLIAM H. TRUEMAN, PHILADELPHIA, PA.

A PAPER READ BEFORE THE PENNSYLVANIA STATE DENTAL SOCIETY, AT CRESSON, PA., JULY 31ST, 1883.

Although I had been quite familiar with the screw as used in regulating teeth for many years, and had made many appliances in which it played an important part, I was not impressed with its great value until I read a paper upon the subject written by Dr. J. N. Farrar (*Dental Cosmos* for Jan., 1878), the second of a series in which he endeavors to reduce the use of the screw in regulating teeth to a system, and to devise a series of appliances that would meet all cases. I think, perhaps, that attempt, and the numerous complicated and delicate devises suggested, has tended to obscure the advantages of the screw as set forth in the paper referred to.

The idea that impressed me so forcibly in Dr. Farrar's paper was the advantage of a *positive* and *intermittent* force in regulating. To me it was new, and until I read it there I have no recollection of ever having seen it mentioned, or ever having seen any appliance made with that end in view. Until then the screw was mostly used to follow up the movement of the teeth, the real moving force being some form of spring, or the force of mastication. It is true there were on the market several forms of steel jack-screws designed for regulating. I have been unable to learn the views of those who invented them, but gather from the advertisements that they were designed simply as a convenient means of applying force, and I think we may safely say that Dr. Farrar deserves the credit of demonstrating that the idea so long held, that the force used in regulating teeth should be a constant and yielding one, of which the spring is a type, was wrong, and that the screw, exerting a positive unyielding and intermittent force, is by far the most effective and least painful.

I do not agree with Dr. Farrar when he limits the movement of the tooth to a small fraction of an inch every twelve hours. I find no rule can be rigidly laid down; it is simply a matter for judgment. A rate of progress that in one case would be excessive, in another would be unnecessarily tedious.

Neither do I agree with him in applying the screw in all cases. There are many that can be more satisfactorily regulated by other means.

My object in bringing this subject before you is to show the advantage of the screw in cases where other means would probably prove cumbersome and inefficient, and to show you that the apparatus may be simple, easily made, easily altered, inexpensive and effective, working rapidly, and with a minimum of pain and inconvenience to the patient.

First, the advantage of the screw. It is a positive motion; if it is moved one quarter of a turn the tooth *must* move a corresponding distance, for it is not dependent upon any action on the part of the patient. It is an unyielding motion; the tooth is moved and held firmly in its new position, and is not disturbed again for hours. That is the secret of its painlessness. It is not like a spring, that, when it has moved the tooth allows some other force to move it back, and thus by a to and fro motion sets up severe irritation. It holds the tooth so firmly that in the most severe cases of interlocking there is no need of capping any of the teeth to keep the jaws apart. It works so rapidly that no injury need be feared from loss of antagonism while the tooth to be moved is taking its new position.

I use the new style steel screw-jack, suggested by Dr. A. McCollum, and kept in stock at the dental depots. It consists of a middle bar which comes in several lengths, with a right and left hand screw and nut at either end. The nuts are intended to rest against the teeth, and are furnished with holes so they can be tied in place, though I do not think that plan a good one. I have found that wherever the steel touches the tooth, whether the contact be a forcible one or not, the enamel is *always* roughened. To avoid that, I always coat with soft solder or tin any part of the steel that may possibly come in contact with the teeth. I desire to lay particular stress on this, as I have seen teeth seriously injured by neglecting it.

The chief difficulty found in using this screw-jack is to hold it firmly in position. It must not only be firm enough to hold its place while at work, but also must be so fixed that the force used to turn it shall not displace it. The force required to turn the screw is far greater than might be expected. No matter how easily the screw may work at first, after it has been in place a short time and becomes oxydized, as it always does, it works stiff, and in some cases the force required to move the tooth is quite formidable, so that both combined make it necessary that the apparatus be held in place firmly. This is important, for if the apparatus is displaced, if it comes off and is immediately replaced, we may expect to lose a day's work. With the screw working so rapidly, repair does not follow the tooth so closely as with slower methods, and as soon as the pressure is released it immediately springs back. I find, as a rule, on replacing the apparatus we can only get the tooth into the position it was before it came off. For the same reason, after the work is done the tooth must be held in its new position more firmly and for a longer time than with other methods. In practice it is best to move the tooth a little beyond the desired position, and to allow the screw to remain in position for a week or more undisturbed, before replacing it with a retaining apparatus.

As each case requires its own special appliances, I can only give an outline of the manner in which the screw is fixed in position.

We first fit a band around the tooth to be moved, generally making it of platina-gold, as with it we obtain the greatest strength with the least bulk. If for one of the front teeth, we make it narrow, fitting it well down on the neck of the tooth, making it spring on tight, so that it cannot possibly slip over the crown. In putting it on, it is not, however, slipped over the crown, but pushed through the little space always found at the necks of the teeth, from the inside of the mouth. It is well to let the ends come well through, and to make them pointed, so that when in place they may be tightened with the pliers, as were the old fashioned narrow bands on plates.

It may require a little tact to adjust it, but the most crowded mouths always have sufficient space for the band at this place. We now select the tooth or teeth to support the fixed end of the screw, and fit to them bands as wide as they will admit of, so shaping them that they will have a tendency to work up into the gum, and after they are fitted, solder on little catches so as to hold them from going too far. We thus obtain a band that will remain firmly in position, It is best to have the band pass around the front of the tooth, and if the teeth are crowded so that it must be made thin, there is a possibility of its being pulled through, which must be guarded against. Where the bicuspids are selected it is best to use two of them; sometimes even three teeth are needed to resist the force used against the tooth to be moved. It is very needful that this end of the screw should be well supported. In some cases it may be necessary for the admission of the bands to press the teeth apart with rubber, but as a rule, if made of thin platina-gold and the edge beveled, they can with a little patience be pushed up into place without separation.

The bands fitted, we solder to each, with silver solder, a tongue of heavy silver plate to support the screw, and while soldering it to the front band extend a tongue to rest upon the palatine surface of the tooth, so that the band shall not press into the gum too hard. These tongues should not conform to the roof of the mouth, but be made straight, otherwise when the tooth moves they will be pressed into the gum.

We are now ready to attach the screw. First enlarge the holes in the bar to at least double their size, so as to admit an instrument strong enough to turn it when in the mouth. Now file the nickel-plating from both nuts, and so shape them that they will fit on the silver tongues snugly and present no sharp edges to the tongue, and then thoroughly coat them with tin, and also tin the lingual surfaces of the silver tongues. Now place the bands in position on the cast, lay the screw with the nuts in position, screwed up nearly as far as they will go, and holding them in place if needful with a little plaster, thoroughly unite the silver tongues and nuts with soft solder ; use it freely and be sure it takes hold well to avoid after trouble. The fixture is now complete. Before putting it in place oil the nuts and screw well; the surplus may be wiped off, but be sure there is plenty between the nut and the screw, or after a few days it will be impossible to turn them. It is often difficult to fix it secure enough to resist the force needed to turn the screw, and it is generally best to support it with the fingers, and to examine closely to see that it is not displaced before dismissing the patient. In screwing up turn gently and slowly and stop for a few moments if it goes hard ; each time screw up as far as the patient can bear, and then from $\frac{1}{8}$ to $\frac{1}{4}$ of a turn more. The pain will cease in a few moments. It is best to go too slow rather than too fast, but that and many other little things are matters for the operator's judgment.

We generally arrange the fixture complete upon the cast, but sometimes it is best to fit the bands to the teeth in the mouth. After they are complete and ready to attach the screw, place them in the mouth and build up plaster in the space between them. We thus obtain a sure guide to get them in the exact position they should occupy.

If it is desired to pull a tooth in, we simply make the bands into loops. If the screw proves too short, change the position of the nuts, or use a longer bar.

It is said that there is danger that an appliance like this will irritate the periosteum, or make mischief by pressing down into the gum, and that there is great danger in moving a tooth so quickly. I admit that all this is true, but presume that those who attempt it are men of intelligence and judgment, and it requires but little of either to avoid any evil result. The inconvenience to the patient is far less than from any form of plate I know of. It is necessary to see the patient at least once a day, for I do not trust them to tighten the screw. I consider it less expense and labor to construct this appliance than to make a vulcanite plate. I have not attempted to explain how every case should be treated, but have simply given a general outline, believing that your own ingenuity will supply all that is omitted.

